# FDG-PET Radiomics for Response Monitoring in Non-Small-Cell Lung Cancer Treated with Radiation Therapy

**DOI:** 10.3390/cancers13040814

**Published:** 2021-02-15

**Authors:** Montserrat Carles, Tobias Fechter, Gianluca Radicioni, Tanja Schimek-Jasch, Sonja Adebahr, Constantinos Zamboglou, Nils H. Nicolay, Luis Martí-Bonmatí, Ursula Nestle, Anca L. Grosu, Dimos Baltas, Michael Mix, Eleni Gkika

**Affiliations:** 1 Department of Radiation Oncology, Division of Medical Physics, University Medical Center Freiburg, Faculty of Medicine, 79106 Freiburg, Germany; tobias.fechter@uniklinik-freiburg.de (T.F.); dimos.baltas@uniklinik-freiburg.de (D.B.); 2 German Cancer Consortium (DKTK), German Cancer Research Center (DKFZ), Partner Site Freiburg of the German Cancer Research Center (DKFZ), 69120 Heidelberg, Germany; Sonia.adebahr@uniklinik-freiburg.de (S.A.); constantinos.zamboglou@uniklinik-freiburg.de (C.Z.); nils.nicolay@uniklinik-freiburg.de (N.H.N.); ursula.nestle@mariahilf.de (U.N.); anca.grosu@uniklinik-freiburg.de (A.L.G.); eleni.gkika@uniklinik-freiburg.de (E.G.); 3 La Fe Health Research Institute, Biomedical Imaging Research Group (GIBI230-PREBI) and Imaging La Fe Node at Distributed Network for Biomedical Imaging (ReDIB) Unique Scientific and Technical Infrastructures (ICTS), 46026 Valencia, Spain; marti_lui@gva.es; 4 Department of Radiation Oncology, University Medical Center Freiburg, Faculty of Medicine, 79106 Freiburg, Germany; gianluca.radiccioni@uniklinik-freiburg.de (G.R.); tanja.schimek-jasch@uniklinik-freiburg.de (T.S.-J.); 5 Department of Radiation Oncology, Kliniken Maria Hilf, GmbH Moenchengladbach, 41063 Moechengladbach, Germany; 6 Department of Nuclear Medicine, University Medical Center Freiburg, Faculty of Medicine, 79106 Freiburg, Germany; michael.mix@uniklinik-freiburg.de

**Keywords:** lung cancer, PET radiomics, FDG monitoring and retrospectively gated 4D PET/CT

## Abstract

**Simple Summary:**

In this study, we strive to identify clinically relevant image feature (IF) changes during chemoradiation in patients with non-small-cell lung cancer (NSCLC) to be able to predict tumor responses in an early stage of treatment. All patients underwent static (3D) and respiratory-gated 4D PET/CT scans before treatment and a 3D scan during or after treatment. Our proposed method rejects IF changes due to intrinsic variability such as noise, resolution and movement through breathing. The IF variability observed across 4D PET is employed as a patient individualized normalization factor to emphasize statistically relevant IF changes during treatment.

**Abstract:**

The aim of this study is to identify clinically relevant image feature (IF) changes during chemoradiation and evaluate their efficacy in predicting treatment response. Patients with non-small-cell lung cancer (NSCLC) were enrolled in two prospective trials (STRIPE, PET-Plan). We evaluated 48 patients who underwent static (3D) and retrospectively-respiratory-gated 4D PET/CT scans before treatment and a 3D scan during or after treatment. Our proposed method rejects IF changes due to intrinsic variability. The IF variability observed across 4D PET is employed as a patient individualized normalization factor to emphasize statistically relevant IF changes during treatment. Predictions of overall survival (OS), local recurrence (LR) and distant metastasis (DM) were evaluated. From 135 IFs, only 17 satisfied the required criteria of being normally distributed across 4D PET and robust between 3D and 4D images. Changes during treatment in the area-under-the-curve of the cumulative standard-uptake-value histogram (δ_AUC_CSH__) within primary tumor discriminated (AUC = 0.87, Specificity = 0.78) patients with and without LR. The resulted prognostic model was validated with a different segmentation method (AUC = 0.83) and in a different patient cohort (AUC = 0.63). The quantification of tumor FDG heterogeneity by δ_AUC_CSH__ during chemoradiation correlated with the incidence of local recurrence and might be recommended for monitoring treatment response in patients with NSCLC.

## 1. Introduction

In non-small cell lung cancer, [^18^F]fluoro-2-deoxy-D-glucose (FDG) PET is a valuable tool for tumor detection and staging [1,2,3,4]. Furthermore, the use of FDG-PET/CT is the standard of care for the definition of the target volume in radiotherapy treatment planning as well as for treatment monitoring [5,6,7,8]. In some studies, relative changes of standardized uptake value (SUV) in lung cancer patients correlated with the treatment outcome [9,10]. Additionally, the feasibility of response assessment during the first weeks of radiotherapy treatment would allow for an adaptation of the treatment strategy, which might lead to better local control rates [11].

Radiomics is the extraction and analysis of a large number of quantitative image features (IF) including first-order (histogram and shape parameters) and second- or higher-order statistics (texture features), which provide spatial and voxel intensity information. Radiomics might help to develop descriptive or predictive models for improving diagnosis or treatment selection [12]. In order to maximize the generalization ability of the resulted model, radiomics requires a high level of IF robustness [13,14,15]. The low spatial resolution and poor statistics of positron emission tomography (PET) images have a negative impact on the IF variability compared to other imaging modalities such as computed tomography (CT). In addition, PET IF variability has been proved to be very sensitive to image reconstruction settings, tumor segmentation methods, SUV resampling methods and texture feature matrix definitions [16,17]. Furthermore, the evaluation of lung cancer lesions using PET/CT imaging presents additional challenges due to respiratory movement. Tumor motion due to breathing cycles during PET scan acquisition results in inaccurate quantification of radioactivity concentration and erroneous estimation of the shape and volume of the lesion. All these degrading factors have an impact on the PET IF variability, from now on referred to as “intrinsic variability”. The effect of each of these degrading factors on IF quality depends on the considered IF. The intrinsic variability does not correlate with clinical changes, that is, physiological processes and if not taken into account leads to a misinterpretation of the results while analyzing clinical data. Thus, there is a need to identify IF variations large enough (clinical) in the quantification of lung lesions changes based on FDG-PET radiomics at different treatment time points to be considered relevant for treatment monitoring [18].

Retrospectively respiratory gated 4D PET/CT has been proposed to minimize respiratory motion degradation in PET/CT systems [19]. Our group previously investigated the IF variability for 3D and 4D PET imaging protocols with experimental heterogeneous phantoms [20] and lung cancer patients [21]. Results showed that although IF variability across the breathing phases depends on the considered IF, all IF followed a normal distribution for most lesions. In addition, and in concordance to previous publications [22], a large number of IF was robust to the motion compensation implied in 4D PET/CT images. The main purpose of this work was to identify relevant changes during chemoradiation of NSCLC lesions based on FDG-PET radiomics and to evaluate the efficacy of radiomics in predicting treatment outcome. Additionally, we aimed to evaluate a novel method, which rejects IF variations originated by the noise, resolution and effects of breathing motion inherent in PET images. The IF variability (standard deviation: σIF) observed across the pre-treatment 4D PET breathing phases was employed as an individualized patient normalization factor to emphasize statistically relevant IF changes during treatment. The prediction of the response was used as a clinical outcome to assess the feasibility of the method. The resultant prognostic model was additionally validated with a different segmentation method and an additional patient cohort.

## 2. Materials and Methods

### 2.1. Patient Cohorts

The entire study cohort consisted of patients with pulmonary lesions derived from two prospective trials (PET Plan and STRIPE). All procedures performed in studies involving human participants were in accordance with the ethical standards of the institutional or national research committee and with the 1964 Helsinki Declaration and its later amendments or comparable ethical standards. The randomized controlled PET-Plan trial (ARO 2009-09, ClinicalTrials.gov Identifier: NCT00697333, Deutsche Krebshilfe, German Cancer Aid Organisation, Nr 108237) was conducted in 24 centers in Germany, Austria and Switzerland in accordance with the Helsinki Declaration. Study design, procedures and main outcome results have been published elsewhere [23]. Briefly, patients with histologically proven inoperable locally advanced NSCLC suitable for chemoradiotherapy were randomly assigned (1:1) to target volume delineation performed on ^18^F-FDG-PET and CT (including CT-positive but ^18^F-FDG-negative nodes) plus elective nodal irradiation and tumor-associated atelectasis, if applicable (conventional target group), or to target volumes defined by PET alone (^18^F-FDG PET-based target group). Patients in the phase II STRIPE trial (Deutsche Krebshilfe, German Cancer Aid Organisation, Nr 108472) with small primary or metastatic lung tumors who refused surgery or whose tumors were inoperable due to comorbidities were treated with SBRT. Study design, procedures and main outcome results have been published elsewhere [24]. The 48 patients in our analyses (37 from PET Plan and 11 from STRIPE) were prospectively recruited in our department.

**Figure 1:**
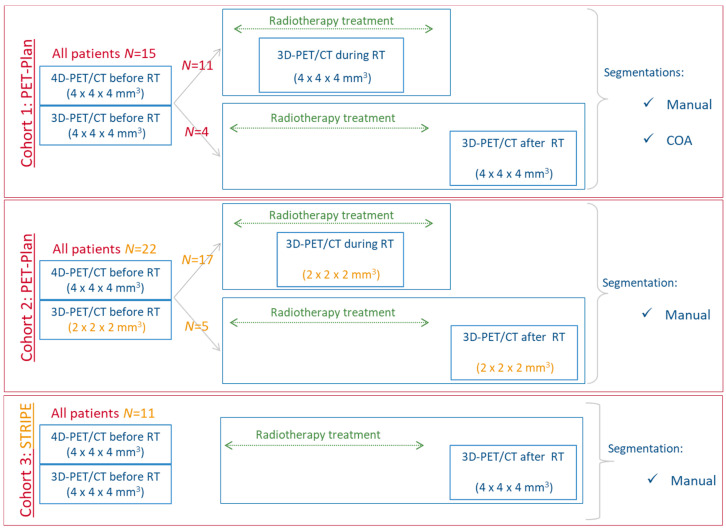
Flowchart of the three cohorts: (i) cohort 1 and 3 were employed to identify the IF satisfying the criteria of being normally distributed across 4D PET and robust between 3D and 4D images; (ii) cohort 1 with manual segmentation of the primary tumor was the training cohort to develop the radiomics model for prediction of treatment response; and (iii) cohort 1 with COA (different segmentation) and cohort 2 (different voxel size for 3D image reconstruction and different patients) to validate the radiomics model.

The main selection criterion for these patients was to have both static 3D PET/CT and retrospectively respiratory-gated 4D PET/CT scans performed before the start of the curatively intended chemoradiotherapy. The study population was divided into three different datasets (Cohorts 1 to 3) based on the number and characteristics of the PET/CT acquisitions. Clinical characteristics of the cohorts used for the development of the prognostic model are summarized in Table 1. Twenty-eight patients (11 from Cohort 1 and 17 from Cohort 2) underwent PET/CT acquisitions during treatment and 20 patients (4 from Cohort 1, 5 from Cohort 2 and 11 from Cohort 3) after treatment (Figure 1). Treatment response was evaluated in terms of overall survival (OS), local recurrence (LR) and distant metastasis (DM) for cohorts 1 and 2.

### 2.2. PET/CT Acquisition

Scans were performed on two different PET/CT systems from Philips (Eindhoven, The Netherlands): GEMINI TF TOF 64 (TF64) and GEMINI TF 16 Big Bore (BB). The scanners fulfilled the requirements indicated in the European Association of Nuclear Medicine (EANM) imaging guidelines (http://earl.eanm.org/ (accessed on 5 February 2021)) and obtained EANM Research Ltd. (EARL) accreditation. The transverse spatial resolution at 1 cm from the central axis of the scanner is 4.8 mm for both scanners. PET data were corrected for randomness, scattering and attenuation based on the corresponding CT dataset. The reconstruction method was a LOR-based ordered-subset iterative time-of-flight algorithm using spherical coordinates (BLOB-OS-TF) with three iterations, 33 subsets and a relaxation parameter of 0.35 for smoothing. Images were normalized to decay-corrected injected activity per kg body weight standardized-uptake-value (SUV) [g/mL]. A 3D PET scan was planned 60 min after the injection of 350 MBq [^18^F]FDG. A 4D PET was acquired after a 3D PET approximately 90 min post injection. The scanning parameters involved in each cohort are summarized in Table 2.

### 2.3. Tumor Segmentation

Two different methods were applied to delineate the primary tumor lesion: (i) a manual contour by consensus of two radiation oncologists and (ii) the semi-automatic segmentation method Contrast-oriented-algorithm (COA) approved by a radiation oncologist. COA was previously validated with heterogeneous experimental phantoms and lung cancer patients [20].

### 2.4. Image Features Extraction

A total of 135 IF were computed with an open-source code [25] based on MATLAB^®^ (The MathWorks Inc., Natick, MA, USA) for all PET images and all segmentations presented in Figure 1. The radiomics IF used in this study are listed in the Supplementary Materials Table S1. As recommended by previous investigations [21,26], SUV values of the voxels within the contour were discretized with a fixed bin width (W = 0.01) for texture feature computation. Texture features were derived from five matrices: the 3D version of the gray-level co-occurrence matrix (GLCM), the gray-level run length matrix (GLRLM), the gray-level size zone matrix (GLSZM) and the neighborhood gray tone difference matrix (NGTDM). In addition, on the voxel intensities within the contour we applied: (i) a Wavelet band-pass filtering (WF) with a weight ratio of 1:2 between band-pass sub-bands and other sub-bands and (ii) an equal-probability quantization algorithm (Q) by using the function histeq of MATLAB^®^.

### 2.5. Statistical Analysis

Statistical analysis was performed using in-house software based on Wolfram Mathematica v 11.2. Normality was evaluated by the Shapiro–Wilk test. The Wilcoxon signed rank (WSR) test was used when comparing two data samples and positive findings were confirmed by the 95% confidence interval (CI) of the Bland–Altman percentage plot analysis [27]. For the analysis of overall survival, Kaplan–Meier curves were estimated and the comparison between groups was evaluated with the log-rank test. Multivariate Cox regression was used for estimation of hazard ratios (HR) with 95% CI. In the analysis of the binary outputs (distant metastasis and local recurrence), the Mann–Whitney U test was used for non-pairwise comparison between groups. For positive findings, an open-source multivariate binary logistic regression analysis [25] was additionally performed that involved imbalance-adjusted bootstrap resampling in prediction performance estimation and the computation of model coefficients. To correct for multiple test comparisons, the *p* values were adjusted for multiple testing by controlling the false discovery rate using Benjamin and Hochberg’s method [28]. A *p* < 0.05 was considered to be statistically significant.

### 2.6. Proposed Method

Our aim was to identify statistically relevant IF variations during chemoradiotherapy under the assumption that they would have a higher probability of being clinically relevant when ruling out those emerging from the intrinsic variability of the PET/CT images. The proposed method is based on two main findings from previous investigations: (i) our group observed that most IF were normally distributed across the breathing phases for 28 heterogeneous phantoms following 16 respiratory patters and for 31 lung cancer patients; (ii) there were IF robust to the motion compensation implied by 4D PET/CT.

**Figure 2:**
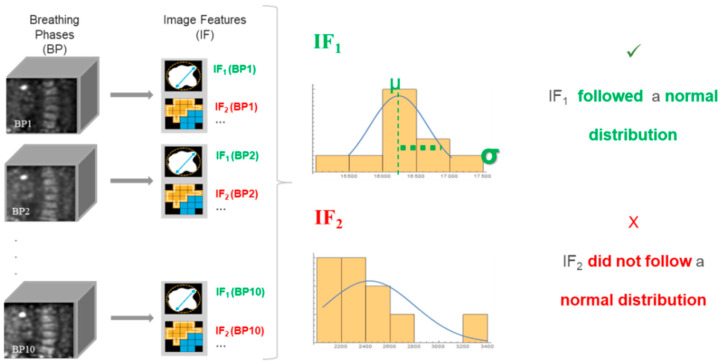
Scheme of the workflow for the first IF selection criteria: IF normal distributed across 4D breathing phases in 70% of the patients.

The method consists of three main steps:

- Identification of the IF following a normal distribution across the pre-treatment 4D breathing phases. The objective was to ensure that the 4D protocol and the robustness of the IF were good enough to reproduce FDG-distribution quantization across the respiratory phases. For each patient, a primary lesion was segmented on each breathing phase and values for the 135 IF were computed, Figure 2. We considered that IF satisfied the selection criteria when their values across the 4D frames followed a normal distribution (Shapiro–Wilk test) in more than 70% of the patients.
- Identification of IF robustness throughout 3D and 4D PET images. The objective was to ensure that the IFs were robust enough to be reproducible with and without a motion-compensation reconstruction protocol.
- Quantification of relative IF variations during treatment ∆r_IF_ weighted according to variability across 4D frames:

(1) $$\delta_{\mathrm{IF}}\;=\;\frac{\Delta r_{\mathrm{IF}}}{\sigma_{\mathrm{IF}}}$$

where this normalization factor permitted the emphasis of statistically relevant IF changes during treatment (δ_IF_).

Consequently, the normalized relative deviation (δ) of the IF, which satisfied both selection criteria, was evaluated for the predictive accuracy of the treatment response.

## 3. Results

### 3.1. Method Development

For the proposed method, the IF that simultaneously satisfied both preconditions—to be normal-distributed along 4D breathing phases and to be robust throughout 3D and 4D PET images—were identified. The results using the Shapiro–Wilk test in the study of normality and the Wilcoxon–Rank test in the study of comparability are summarized in Table 3. The names of the IF are listed in the Supplementary Material in Table S2. Overall, 17 IF were simultaneously normal-distributed along 4D breathing phases and robust throughout 3D and 4D PET images (definitions in Table S1): AUC_CSH_, Variance_CM_, WF-Variance_CM_, WF-SRE,WF-LRE, WF-RLN, WF-RP, Q-SZE, Q-LZE, Q-ZSN, Q-ZP, Q-SRE, Q-LRE, Q-RLN, Q-RP, Q-Contrast_NG_, Q-Busyness.

### 3.2. Prognostic Model

Cohort 1 and cohort 2 were used for the development of the prognostic model. Both prospective cohorts presented similar average values for overall survival (OS): 47 ± 36 months for cohort 1 and 47 ± 21 months for cohort 2. Local recurrence (LR) was observed in 6 out of 15 patients (40%) from cohort 1 and in 9 out of 22 (41%) in cohort 2. Distant metastases (DM) were observed in 6 out of 15 patients (40%) in cohort 1 and in 15 out of 22 (68%) in cohort 2.

We evaluated the prediction of response for the normalized relative deviation of the 17 IF derived from the evaluation in the above-mentioned method. The analysis was initially carried out for cohort 1 using a manual segmentation and sequentially validated in cohort 1 with COA segmentation and in cohort 2 using manual segmentation. Statistically significant correlations between δ_IF_ and treatment outcome were considered false positives, that is, casually linked with the outcome if they were not significant for all (training and validation) cohorts. The only IF that showed statistically significant correlation with the treatment outcome for all cohorts was the area-under-the curve of the cumulative histogram (AUC_CSH_). The normalized relative deviation of AUC_CSH_ (δ_AUC_CSH__) could differentiate patients with LR from patients without LR in patients with locally advanced NSCLC treated with chemoradiation.

**Figure 3:**
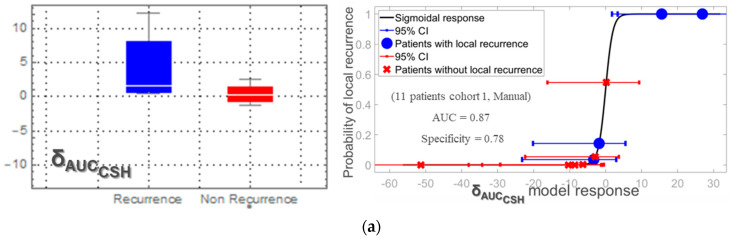

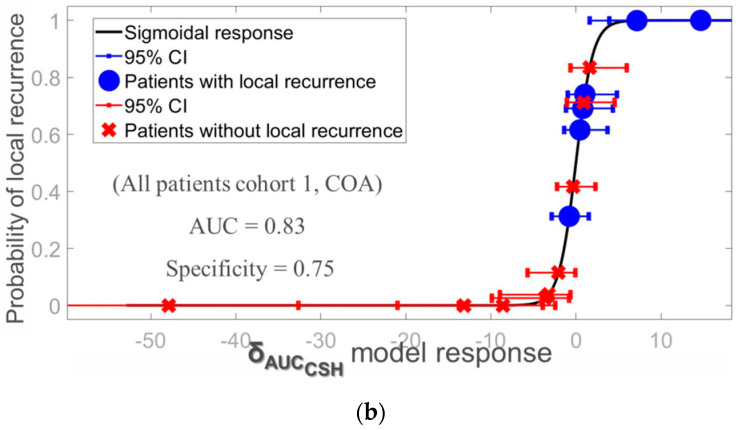
Prediction of patients with local recurrence based on AUC_CSH_ heterogeneity quantification for patients of cohort 1 with 3D PET during RT (*N* = 11) and lesions segmented manually (**a**) and for all patients in cohort 1, that is, including patients with 3D PET after RT and lesions segmented by COA (**b**).

**Figure 4:**
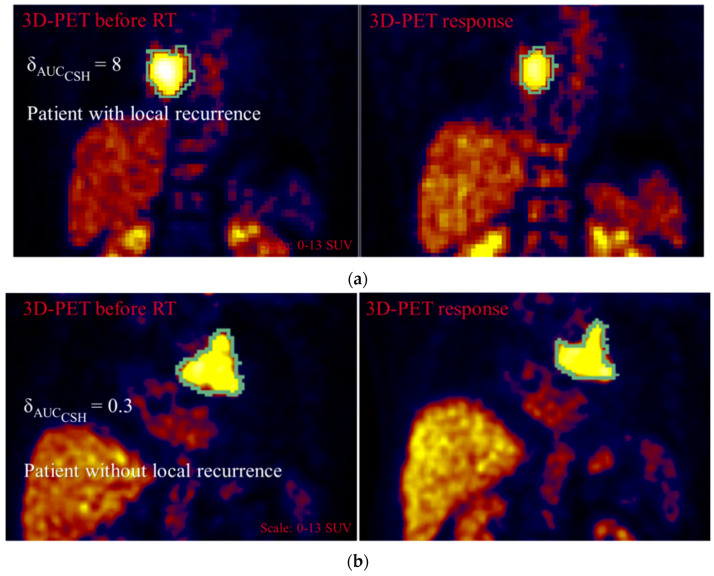
Example of 3D PET images for patient with (**a**) and without (**b**) local recurrence.

In Figure 3a results for the manual segmentation was applied for the 11 patients of cohort 1 with 3D PET during radiotherapy (RT) are shown. The time interval between the 3D PET images (before and during RT) was 19 ± 10 days. The resulted prognostic model had an AUC of 0.87 and a specificity of 0.78. δ_AUC_CSH__ resulted in a median = 5, mean ± standard deviation = 7.2 ± 4.5 and range of (1.3, 12.3) for patients with LR, and in median = 0.3, mean ± standard deviation = 0.8 ± 5.9 and range of (−16, 2.6) for patients without LR. Therefore, patients with increasing homogeneity in the primary tumor during chemoradiation had a higher probability of local recurrence. Results were confirmed when all 15 patients of cohort 1 were considered, including those with FDG-PET after the end of RT. For these 15 patients the average time interval between treatment and response FDG PET was 68 ± 92 days. The accuracy in RL discrimination showed an AUC = 0.80 and specificity = 0.74. Similar accuracy results (AUC = 0.83 and specificity = 0.75) were obtained when COA was employed instead of the manual segmentation (Figure 3b). Lower model accuracy (AUC = 0.63 and specificity = 0.61) was obtained for the 22 additional patients from cohort 2 with manual segmentation. An example of 3D PET images with and without LR is presented in Figure 4.

## 4. Discussion

Our novel method based on FDG-PET radiomics allows identifying clinically relevant radiomic changes during chemoradiation in locally advanced inoperable NSCLC. We could confirm that 17 IF satisfied simultaneously the two criteria required for the feasibility of the method: they were normal-distributed across 4D breathing frames, and they were consistent throughout 3D and 4D images. Consequently, for these 17 IFs we could justify the use of the variability across the pre-treatment 4D PET breathing phases as a patient individualized normalization factor to emphasize statistically relevant IF changes during treatment. Furthermore, we tested the implementation of the method in the prediction of treatment outcome in 37 lung cancer patients and preliminary validation results showed that the normalized relative deviation of the AUC of the cumulative histogram, δ_AUC_CSH__, differentiates patients with local recurrence from patients without local recurrence. 

In the development of the method, different segmentation approaches and patient cohorts were used. When comparing the same cohort but with different segmentation approaches, manual segmentation resulted in a larger number of IF satisfying the two criteria (65 normal-distributed and 83 comparable) than for COA (61 normal-distributed and 69 comparable). The decreasing number of robust IF for COA could be justified by the fact that, in comparison with manual segmentation, automatic segmentations like COA are more sensitive to image noise, heterogeneity and signal blurring due to the lesion motion [20]. When comparing two different patient cohorts, more IF were robust between 3D and 4D in cohort 3 (131 IF) than for the same segmentation in cohort 1 (83 IF). It could not be explained by the range of lesion sizes or locations involved in the cohorts because decreased robustness would be expected for smaller lesions and for more significant lesion movement. However, in cohort 3 the lesions were smaller (median volume = 12 ± 9 mL) and 7/11 had a peripheral location compared to cohort 1 where lesions were larger (median volume = 86 ± 66 mL) and 3/15 were peripheral. A possible explanation for the unexpected decreased number of robust IF observed for cohort 1 could be a more irregular respiratory patterns for these central lesions as the respiratory pattern is a very significant degrading factor for IF robustness between 4D and 3D [29]. Overall, IF variability was sensitive to the segmentation and to the localization and volume of the treated lesions. We could therefore emphasize the importance of involving different cohorts in model development in order to increase the ability to generalize the resulting method. 

In addition, it should be remarked that the method proposed would permit the identification of clinically relevant changes during treatment for a given image feature. In previous publications, two approaches were employed to define PET IF stability: In first method, IF were considered not stable if their relative deviations were larger than the relative deviations observed for volume or for SUV (maximum and mean) [30]. The second method was to consider IF as stable if they had a relative deviation lower than 15% [31] Although these methods were considered reasonable, it has been demonstrated that for phantoms [29] and for patients [21] the intrinsic variability of an IF and the impact of the degrading factor on an IF [16,32] varies between the respective features. Consequently, it should be recommended that a stability criterion individually adapted to the respective IF be used and the use of general rules be avoided. In addition, the criteria to identify clinically relevant changes during treatment using the proposed method would be also patient-dependent. It is well known that PET noise and resolution are degrading factors of IF variability and their impact on IF variability can be expected to be more significant for smaller lesions and lower uptake concentrations. Therefore, a stability criterion individually adapted to the patient, that is, adapted to the SUV and volume of the lesion, would be preferable to a common stability criterion applied to all patients independent of lesions characteristics. 

Once we identified the IF eligible for their use in the proposed method we tested the implementation of the method in the outcome prediction for lung cancer patients, who were separated into training cohorts and validation cohorts. The training cohort was selected in terms of homogeneity: all response 3D PETs were acquired in the second week after the start of the treatment, and all PETs had the same resolution. Interestingly, accuracy obtained for the model of LR prediction in the training cohort (AUC = 0.87, Specificity = 0.78) was also high (AUC = 0.80, specificity = 0.74) when 4 additional patients, for whom response 3D PET was acquired after treatment, were included. For the validation cohorts, the model accuracy was independent on whether 3D PET was done during or after treatment. These results would not be enough to confirm the robustness of the method with respect to the time of the 3D PET acquisition for response monitoring. However, based on these results, the robustness of the method with respect to the time could not be rejected, which would imply an important advantage for the application of the method in clinical routine. In addition, LR prediction was equally accurate for lesions segmented manually or by COA. From these results, the replacement of manual segmentation by COA segmentation could be recommended. It would not only reduce inter- and intra-observer variability, but it would also reduce the time invested, which is requisite for the application of the method in clinical routine. Although previous phantom experiments had already reported the robustness of AUC_CSH_ with respect to different PET/CT systems, CT metal artefacts and reconstruction voxel size [32], the accuracy of the LR prediction model was also evaluated for a second validation cohort of 22 new patients, mixing 2 and 4 mm PET images. For cohort 2, accuracy results were poorer but still comparable to findings reported in previous publications [33].

Many publications focused on radiomics in lung cancer [34,35,36], but only part of these publications focused on FDG-PET images. From them, a small number evaluated IF variations during or after treatment (delta radiomics) [33]. To our knowledge, we present the first prediction model based on IF variations adjusted to account for the intrinsic variability of IF. Our results showed that δ_AUC_CSH__ could differentiate patients with local recurrence from patients without local recurrence. AUC_CSH_ is a first-order statistics, that is, it does not take into account spatial information, which reflects tumor heterogeneity. The cumulative SUV-volume histogram (CSH) is an analogue to the dose-volume histogram employed in RT-planning. In CSH, the percent of tumor volume with an SUV (instead of dose) above a certain threshold is plotted against that threshold, which varied from 0 to 100% of the maximum SUV (SUV_max_). The area under the curve of these plots, AUC_CSH_, is a quantitative index of heterogeneity, where AUC_CSH_ increases with homogeneity. For patients with local recurrence δ_AUC_CSH__ resulted in (median = 5, mean ± standard deviation = 7.2 ± 4.5, ranging from 1.3 to 12.3) and for patients without local recurrence in (median = 0.3, mean ± standard deviation = 0.8 ± 5.9, ranging from −16 to 2.6) (See example in Figure 2). We could, therefore, conclude that patients whose primary tumor homogeneity increased during therapy had a higher probability of local recurrence. Previous publications have also reported significant correlations between AUC_CSH_ and RT outcome for lung cancer patients: prediction of recurrence based on pre-treatment AUC_CSH_ quantification [37] and prediction of OS based on changes in AUC_CSH_ during treatment [38]. The same correlations could be observed in our data sample, but they were not statistically significant. Although it is known that intratumoral heterogeneity in FDG distribution correlates with many factors at the cellular level (such as intracellular hypoxia, necrotic infiltration, vascularization and tumor cell proliferation [39]), the underlying physiological processes, which might explain why an increased FDG homogeneity in terms of AUC would be derived in local recurrence are still not clear. For a better understanding of our results on a cellular or immunological level (e.g., by an increase in the T-cell infiltration), it would be of interest to evaluate our findings with a mid-treatment histological examination. From our knowledge, no publication concerning other cancer sites has demonstrated a statistically significant prediction of treatment response by AUC_CSH_. It could probably be due to the fact that AUC_CSH_ is not as frequently evaluated as other histogram parameters [16], such as SUV_mean_, Skewness, Kurtosis or coefficient of variance. Nevertheless, patients with an increase in primary tumor homogeneity during therapy (e.g., diagnosed using a mid-treatment ^1^⁸F-FDG PET) might benefit from concepts such as dose escalation or an additional boost of the primary tumor. In order to avoid treatment prolongation and subsequently avoiding the effect of an accelerated repopulation of tumor clonogens [40,41], either hyperfractionation [42] or hypofractionation [11] can be used. Such concepts have been tested previously, using metabolic tumor volumes according to ^1^⁸F-FDG-avidity on mid-treatment PET scans with conflicting results [42,43,44]. From the results reported in this study, it might be of interest to evaluate these treatments on the basis of an increase of ^1^⁸F-FDG homogeneity in terms of AUC. 

The main limitation of our study is the small sample size. A wide patient population is required to increase the statistical significance of our results. Current work is therefore focused on the recruitment of additional patients from other German institutions involved in STRIPE and PET-Plan trials. This wider, more heterogeneous, patient population will allow us to evaluate the performance of our model in an external validation cohort. Consequently, the LR prediction model observed for the patient population involved in the current analysis should be understood as a proof of concept for the feasibility of the use of the proposed method to identify clinical relevant changes during the treatment of lung cancer patients based FDG-PET radiomics. The confirmation of this LR prediction in a larger patient cohort is therefore required. However, from our preliminary results, and in agreement with previous publications, the quantification of tumor FDG heterogeneity by δ_AUC_CSH__ could be implemented when monitoring treatment response of NSCLC patients treated with chemoradiation and assist with mid-treatment adaption and dose intensification concepts such as isotopic dose escalation.

## 5. Conclusions

The presented novel method based on FDG-PET radiomics identifies clinically relevant changes during chemoradiation in patients with NSCLC. For the selected 17 IF, which were normally distributed across 4D breathing frames and robust throughout 3D and 4D images, we could justify the use of the variability across the pre-treatment 4D PET breathing phases as a patient-individualized normalization factor to emphasize statistically relevant IF changes during treatment. In addition, we reported the first prediction model based on IF variations which were adjusted to account for the intrinsic variability of IF. Preliminary results showed that patients for which primary tumor homogeneity, quantified by AUC_CSH_, increased during therapy had a higher probability for local recurrence. These patients could profit the most from mid-treatment adaptions of the target volume as well as from isotoxically escalated concepts such as in the PET-Plan trial.

## Figures and Tables

**Table 1:** Clinical characteristics for the patient cohorts used in this study.

Clinical Characteristics	Cohort 1 (*n* = 15)	Cohort 2 (*n* = 22)	Cohort 3 (*n* = 11)
Clinical Trial Register	NCT00697333	NCT00697333	DRKS00003658
	PET-Plan	PET-Plan	STRIPE
Age (years, mean ± SD, range)	(67 ± 9, 51–78)	(65 ± 10, 47–83)	(74 ± 7, 60–83)
Sex	-	-	-
Female	9(60%)	6(27%)	3(27%)
Male	6(40%)	16(73%)	8(73%)
Tumor Localization	-	-	-
Peripheral	3(20%)	7(32%)	7(73%)
Central	12(80%)	15(68%)	3(27%)
Stage	-	-	-
Ib	(0%)	(0%)	6(55%)
IIb	(0%)	2(9%)	1(9%)
IIIa	8(53%)	5(23%)	(0%)
IIIb	5(33%)	9(41%)	(0%)
IIIc	1(7%)	5(23%)	(0%)
IV	(0%)	1(5%)	2(18%)
Metastases	(0%)	(0%)	2(18%)
Chemotherapy	-	-	-
Concurrent	15(100%)	22(100%)	(0%)
No	(0%)	(0%)	11(100%)
Radiotherapy	(65 ± 11, 30–74)	(66 ± 5, 60–74)	(36 ± 1, 35–38) *
(Gy, mean ± SD, range)	-	-	-
Overall Survival	(47 ± 36, 1–105)	(47 ± 21,4–77)	(60 ± 31, 11–105)
(months, mean ± SD, range)	-	-	-
Local Recurrence (yes)	6(40%)	9(41%)	2(18%)
Distant Metastasis (yes)	6(40%)	15(68%)	6(55%)

SD: standard deviation. * Prescribed dose in 60% Isodose.

**Table 2:** PET scanning parameters.

Scannig Parameters	Cohort 1 (*n* = 15)	Cohort 2 (*n* = 22)	Cohort 3 (*n* = 11)
4D PET/CT before RT	yes	yes	yes
PET/CT System	-	-	-
TF-64	15	4	11
BB	0	18	0
Voxel Dimension (mm^3^)	4 × 4 × 4	4 × 4 × 4	4 × 4 × 4
3DPET/CT before RT	yes	yes	yes
PET/CT System	-	-	-
TF-64	15	4	11
BB	0	18	0
Voxel Dimension (mm^3^)	4 × 4 × 4	2 × 2 × 2	4 × 4 × 4
3DPET/CT during RT	yes	yes	yes
PET/CT System	-	-	-
TF-64	11	4	11
BB	0	13	0
Voxel Dimension (mm^3^)	4 × 4 × 4	2 × 2 × 2	4 × 4 × 4
3DPET/CT after RT	yes	yes	yes
PET/CT System	-	-	-
TF-64	4	0	11
BB	0	5	0
Voxel Dimension (mm^3^)	4 × 4 × 4	4 × 4 × 4	4 × 4 × 4
Time interval between 3D scans (days, mean ± SD, range)	(68 ± 92, 14–343)	(196 ± 340, 13–1123)	(140 ± 67, 42–271)

**Table 3:** Number of IF in each cohort and for each segmentation (manual or contrast-oriented algorithm COA) that satisfied the criteria required for the method proposed.

Segmentation	Cohort 1 Manual	Cohort 1 COA	Cohort 3 Manual	All
Normal Distributed across 4D	65	61	50	31
Comparable (4D vs. 3D)	83	69	131	62

## Data Availability

Data sharing not applicable.

## Supplementary Materials

The following are available online at https://www.mdpi.com/2072-6694/13/4/814/s1. Table S1: Image features (radiomics) and Table S2: Image features robustness required for the proposed method. Black box means positive result for the analysis described on the first row and represents the property of interest. In last column, IF robustness was defined by simultaneously satisfying both criteria (normal-distributed (fifth column) and comparable (ninth column)).
