# Supplementary material for: FDG-PET Radiomics for Response Monitoring in Non-Small-Cell Lung Cancer Treated with Radiation Therapy
Source: Cancers (Basel). 2021 Feb 15;13(4):814. doi: 10.3390/cancers13040814 (PMC7919471; doi:10.3390/cancers13040814)
Supplement: Supplementary file 1 [file cancers-13-00814-s001.zip › SM_Tables/ESM_Table2.docx]

**ESM Table 2.** Image features robust required for the proposed method. Black box means positive result for the analysis described on the first row and represents the property of interest. In last column, IF robust were defined by simultaneously satisfying both criteria (normal distributed (fifth column) and comparable (ninth column).

|  | **Normal Distributed across 4D** | | | | **Comparable (4D vs 3D)** | | | |  |
| --- | --- | --- | --- | --- | --- | --- | --- | --- | --- |
|  | Cohort1:Manual | Cohort1:COA | Cohort2:Manual | All | Cohort1:Manual | Cohort1:COA | Cohort2:Manual | All | **Robust** |
| **Image Features** | 65 | 61 | 50 | 31 | 83 | 69 | 131 | 62 | **17** |
| V | 0 | 0 | 0 | 0 | 1 | 1 | 1 | 1 | 0 |
| SUV_max_ | 0 | 0 | 0 | 0 | 0 | 0 | 1 | 0 | 0 |
| TL-FET | 0 | 0 | 0 | 0 | 0 | 0 | 1 | 0 | 0 |
| SUV_peak_ | 0 | 0 | 0 | 0 | 0 | 0 | 1 | 0 | 0 |
| SUV_mean_ | 1 | 0 | 0 | 0 | 0 | 0 | 1 | 0 | 0 |
| AUC_CSH_ | 1 | 1 | 1 | 1 | 1 | 1 | 1 | 1 | 1 |
| SUVmin | 1 | 0 | 1 | 0 | 1 | 0 | 1 | 0 | 0 |
| CoV | 0 | 1 | 1 | 0 | 0 | 1 | 1 | 0 | 0 |
| Skewness | 0 | 1 | 1 | 0 | 1 | 1 | 0 | 0 | 0 |
| Kurtosis | 0 | 1 | 1 | 0 | 1 | 1 | 0 | 0 | 0 |
| EntropyLog2 | 0 | 1 | 0 | 0 | 0 | 1 | 1 | 0 | 0 |
| Energy (E_H_) | 1 | 0 | 0 | 0 | 1 | 0 | 1 | 0 | 0 |
| IU | 1 | 1 | 1 | 1 | 0 | 1 | 1 | 0 | 0 |
| DU | 0 | 0 | 0 | 0 | 1 | 1 | 1 | 1 | 0 |
| Solidity | 0 | 0 | 1 | 0 | 1 | 1 | 1 | 1 | 0 |
| Eccentricity | 1 | 0 | 1 | 0 | 1 | 1 | 0 | 0 | 0 |
| LD | 0 | 0 | 0 | 0 | 1 | 1 | 1 | 1 | 0 |
| PI | 0 | 0 | 0 | 0 | 1 | 1 | 1 | 1 | 0 |
| Energy_CM_ | 0 | 0 | 0 | 0 | 1 | 1 | 1 | 1 | 0 |
| Contrast_CM_ | 0 | 0 | 0 | 0 | 0 | 0 | 1 | 0 | 0 |
| Entorpy_CM_ | 0 | 0 | 0 | 0 | 1 | 1 | 1 | 1 | 0 |
| LH | 1 | 0 | 0 | 0 | 0 | 0 | 1 | 0 | 0 |
| Correlation_CM_ | 0 | 1 | 0 | 0 | 1 | 1 | 1 | 1 | 0 |
| Variance_CM_ | 1 | 1 | 1 | 1 | 1 | 1 | 1 | 1 | 1 |
| D | 0 | 0 | 0 | 0 | 0 | 0 | 1 | 0 | 0 |
| Acor | 0 | 0 | 0 | 0 | 0 | 0 | 1 | 0 | 0 |
| SZE | 1 | 1 | 1 | 1 | 0 | 0 | 1 | 0 | 0 |
| LZE | 1 | 1 | 1 | 1 | 0 | 0 | 1 | 0 | 0 |
| GLN | 0 | 0 | 0 | 0 | 1 | 1 | 1 | 1 | 0 |
| ZSN | 1 | 1 | 1 | 1 | 0 | 0 | 1 | 0 | 0 |
| ZP | 1 | 1 | 1 | 1 | 0 | 0 | 1 | 0 | 0 |
| LGZE | 0 | 1 | 1 | 0 | 1 | 0 | 1 | 0 | 0 |
| HGZE | 0 | 0 | 0 | 0 | 0 | 0 | 1 | 0 | 0 |
| SZLGE | 0 | 1 | 1 | 0 | 1 | 0 | 1 | 0 | 0 |
| SZHGE | 0 | 0 | 0 | 0 | 0 | 0 | 1 | 0 | 0 |
| LZLGE | 0 | 1 | 1 | 0 | 1 | 0 | 1 | 0 | 0 |
| LZHGE | 0 | 0 | 0 | 0 | 0 | 0 | 1 | 0 | 0 |
| GLV | 0 | 0 | 0 | 0 | 1 | 0 | 1 | 0 | 0 |
| ZSV | 0 | 0 | 0 | 0 | 0 | 1 | 1 | 0 | 0 |
| SRE | 1 | 1 | 1 | 1 | 0 | 0 | 1 | 0 | 0 |
| LRE | 1 | 1 | 1 | 1 | 0 | 0 | 1 | 0 | 0 |
| GLN2 | 0 | 0 | 0 | 0 | 1 | 1 | 1 | 1 | 0 |
| RLN | 1 | 1 | 1 | 1 | 0 | 0 | 1 | 0 | 0 |
| RP | 1 | 1 | 1 | 1 | 0 | 0 | 1 | 0 | 0 |
| LGRE | 0 | 1 | 1 | 0 | 1 | 0 | 1 | 0 | 0 |
| HGRE | 0 | 0 | 0 | 0 | 0 | 0 | 1 | 0 | 0 |
| SRLGE | 0 | 1 | 1 | 0 | 1 | 0 | 1 | 0 | 0 |
| SRHGE | 0 | 0 | 0 | 0 | 0 | 0 | 1 | 0 | 0 |
| LRLGE | 0 | 1 | 1 | 0 | 1 | 0 | 1 | 0 | 0 |
| LRHGE | 0 | 0 | 0 | 0 | 0 | 0 | 1 | 0 | 0 |
| GLV2 | 1 | 0 | 0 | 0 | 1 | 0 | 1 | 0 | 0 |
| RLV | 0 | 0 | 0 | 0 | 0 | 0 | 1 | 0 | 0 |
| Coarseness | 0 | 1 | 1 | 0 | 0 | 0 | 1 | 0 | 0 |
| Contrast_NM_ | 0 | 0 | 1 | 0 | 0 | 0 | 0 | 0 | 0 |
| Busyness | 1 | 0 | 0 | 0 | 0 | 0 | 1 | 0 | 0 |
| Complexity | 0 | 0 | 0 | 0 | 0 | 0 | 1 | 0 | 0 |
| TS | 0 | 0 | 0 | 0 | 0 | 0 | 1 | 0 | 0 |
| WF_E_CM_ | 0 | 0 | 0 | 0 | 1 | 1 | 1 | 1 | 0 |
| WF_Con_CM_ | 0 | 0 | 0 | 0 | 0 | 0 | 1 | 0 | 0 |
| WF_Ent_CM_ | 0 | 0 | 0 | 0 | 1 | 1 | 1 | 1 | 0 |
| WF_LH | 1 | 1 | 1 | 1 | 0 | 0 | 1 | 0 | 0 |
| WF_C_CM_ | 1 | 1 | 0 | 0 | 1 | 1 | 1 | 1 | 0 |
| WF_Var_CM_ | 1 | 1 | 1 | 1 | 1 | 1 | 1 | 1 | 1 |
| WF_D | 0 | 0 | 0 | 0 | 0 | 0 | 1 | 0 | 0 |
| WF_Acor | 1 | 0 | 0 | 0 | 0 | 0 | 1 | 0 | 0 |
| WF_SZE | 1 | 1 | 1 | 1 | 1 | 0 | 1 | 0 | 0 |
| WF_LZE | 1 | 1 | 1 | 1 | 1 | 0 | 1 | 0 | 0 |
| WF_GLN | 0 | 0 | 0 | 0 | 1 | 1 | 1 | 1 | 0 |
| WF_ZSN | 1 | 1 | 1 | 1 | 1 | 0 | 1 | 0 | 0 |
| WF_ZP | 1 | 1 | 1 | 1 | 1 | 0 | 1 | 0 | 0 |
| WF_LGZE | 0 | 0 | 0 | 0 | 1 | 1 | 1 | 1 | 0 |
| WF_HGZE | 1 | 0 | 0 | 0 | 0 | 0 | 1 | 0 | 0 |
| WF_SZLGE | 0 | 0 | 0 | 0 | 1 | 1 | 1 | 1 | 0 |
| WF_SZHGE | 1 | 0 | 0 | 0 | 0 | 0 | 1 | 0 | 0 |
| WF_LZLGE | 0 | 0 | 0 | 0 | 1 | 1 | 1 | 1 | 0 |
| WF_LZHGE | 1 | 0 | 0 | 0 | 0 | 0 | 1 | 0 | 0 |
| WF_GLV | 0 | 0 | 0 | 0 | 1 | 0 | 1 | 0 | 0 |
| WF_ZSV | 0 | 0 | 0 | 0 | 1 | 0 | 1 | 0 | 0 |
| WF_SRE | 1 | 1 | 1 | 1 | 1 | 1 | 1 | 1 | 1 |
| WF_LRE | 1 | 1 | 1 | 1 | 1 | 1 | 1 | 1 | 1 |
| WF_GLN2 | 0 | 0 | 0 | 0 | 1 | 1 | 1 | 1 | 0 |
| WF_RLN | 1 | 1 | 1 | 1 | 1 | 1 | 1 | 1 | 1 |
| WF_RP | 1 | 1 | 1 | 1 | 1 | 1 | 1 | 1 | 1 |
| WF_LGRE | 0 | 0 | 0 | 0 | 1 | 1 | 1 | 1 | 0 |
| WF_HGRE | 1 | 0 | 0 | 0 | 0 | 0 | 1 | 0 | 0 |
| WF_SRLGE | 0 | 0 | 0 | 0 | 1 | 1 | 1 | 1 | 0 |
| WF_SRHGE | 1 | 0 | 0 | 0 | 0 | 0 | 1 | 0 | 0 |
| WF_LRLGE | 0 | 0 | 0 | 0 | 1 | 1 | 1 | 1 | 0 |
| WF_LRHGE | 1 | 0 | 0 | 0 | 0 | 0 | 1 | 0 | 0 |
| WF_GLV2 | 0 | 0 | 0 | 0 | 1 | 0 | 1 | 0 | 0 |
| WF_RLV | 0 | 0 | 0 | 0 | 0 | 0 | 1 | 0 | 0 |
| WF_Coar | 0 | 0 | 0 | 0 | 0 | 0 | 1 | 0 | 0 |
| WF_Con_NM_ | 0 | 0 | 1 | 0 | 0 | 0 | 1 | 0 | 0 |
| WF_B | 1 | 0 | 0 | 0 | 0 | 0 | 1 | 0 | 0 |
| WF_Comp | 0 | 1 | 0 | 0 | 0 | 0 | 1 | 0 | 0 |
| WF_TS | 0 | 0 | 0 | 0 | 0 | 0 | 1 | 0 | 0 |
| QEnergy_CM_ | 0 | 1 | 1 | 0 | 1 | 1 | 1 | 1 | 0 |
| QContrast_CM_ | 1 | 1 | 0 | 0 | 1 | 1 | 1 | 1 | 0 |
| QEntorpy_CM_ | 0 | 0 | 0 | 0 | 1 | 1 | 1 | 1 | 0 |
| QLH | 1 | 1 | 0 | 0 | 1 | 1 | 1 | 1 | 0 |
| QC_CM_ | 1 | 1 | 0 | 0 | 1 | 1 | 1 | 1 | 0 |
| QVariance_CM_ | 0 | 0 | 1 | 0 | 0 | 0 | 1 | 0 | 0 |
| QD | 1 | 1 | 0 | 0 | 1 | 1 | 1 | 1 | 0 |
| QAcor | 1 | 1 | 0 | 0 | 1 | 1 | 1 | 1 | 0 |
| QSZE | 1 | 1 | 1 | 1 | 1 | 1 | 1 | 1 | 1 |
| QLZE | 1 | 1 | 1 | 1 | 1 | 1 | 1 | 1 | 1 |
| QGLN | 0 | 1 | 0 | 0 | 1 | 1 | 1 | 1 | 0 |
| QZSN | 1 | 1 | 1 | 1 | 1 | 1 | 1 | 1 | 1 |
| QZP | 1 | 1 | 1 | 1 | 1 | 1 | 1 | 1 | 1 |
| QLGZE | 0 | 0 | 0 | 0 | 1 | 1 | 1 | 1 | 0 |
| QHGZE | 1 | 1 | 0 | 0 | 1 | 1 | 1 | 1 | 0 |
| QSZLGE | 1 | 0 | 0 | 0 | 1 | 1 | 1 | 1 | 0 |
| QSZHGE | 1 | 1 | 0 | 0 | 1 | 1 | 1 | 1 | 0 |
| QLZLGE | 0 | 0 | 0 | 0 | 1 | 1 | 1 | 1 | 0 |
| QLZHGE | 1 | 1 | 0 | 0 | 1 | 1 | 1 | 1 | 0 |
| QGLV | 1 | 0 | 1 | 0 | 1 | 0 | 1 | 0 | 0 |
| QZSV | 1 | 1 | 0 | 0 | 0 | 0 | 1 | 0 | 0 |
| QSRE | 1 | 1 | 1 | 1 | 1 | 1 | 1 | 1 | 1 |
| QLRE | 1 | 1 | 1 | 1 | 1 | 1 | 1 | 1 | 1 |
| QGLN2 | 0 | 0 | 0 | 0 | 1 | 1 | 1 | 1 | 0 |
| QRLN | 1 | 1 | 1 | 1 | 1 | 1 | 1 | 1 | 1 |
| QRP | 1 | 1 | 1 | 1 | 1 | 1 | 1 | 1 | 1 |
| QLGRE | 1 | 0 | 0 | 0 | 1 | 1 | 1 | 1 | 0 |
| QHGRE | 1 | 1 | 0 | 0 | 1 | 1 | 1 | 1 | 0 |
| QSRLGE | 1 | 0 | 0 | 0 | 1 | 1 | 1 | 1 | 0 |
| QSRHGE | 1 | 1 | 0 | 0 | 1 | 1 | 1 | 1 | 0 |
| QLRLGE | 1 | 0 | 0 | 0 | 1 | 1 | 1 | 1 | 0 |
| QLRHGE | 1 | 1 | 0 | 0 | 1 | 1 | 1 | 1 | 0 |
| QGLV2 | 1 | 0 | 1 | 0 | 0 | 0 | 1 | 0 | 0 |
| QRLV | 0 | 0 | 0 | 0 | 0 | 0 | 1 | 0 | 0 |
| QCoar | 0 | 1 | 0 | 0 | 1 | 1 | 1 | 1 | 0 |
| QCont_NM_ | 1 | 1 | 1 | 1 | 1 | 1 | 1 | 1 | 1 |
| QB | 1 | 1 | 1 | 1 | 1 | 1 | 1 | 1 | 1 |
| QComp | 0 | 0 | 0 | 0 | 1 | 1 | 1 | 1 | 0 |
| QTS | 0 | 1 | 0 | 0 | 1 | 1 | 1 | 1 | 0 |
